# Gammaherpesviruses and canine lymphoma: no evidence for direct involvement in commonly occurring lymphomas

**DOI:** 10.1099/vir.0.000106

**Published:** 2015-07

**Authors:** Elspeth M. Waugh, Alice Gallagher, Karen A. McAulay, Joaquim Henriques, Margarida Alves, Adam J. Bell, Joanna S. Morris, Ruth F. Jarrett

**Affiliations:** ^1^​ MRC-University of Glasgow Centre for Virus Research, Institute of Infection, Immunity and Inflammation, College of Medical, Veterinary and Life Sciences, 464 Bearsden Road, Glasgow G61 1QH, UK; ^2^​ Centro Veterinario Berna, Av. Berna 35C, 1050-038 Lisbon, Portugal; ^3^​ Centro de Investigação em Biociências e Tecnologias da Saúde (CBiOS), Faculdade de Medicina Veterinária (FMV)/Universidade Lusófona de Humanidades e Tecnologias (ULHT), Lisbon, Portugal; ^4^​ School of Veterinary Medicine, College of Medical, Veterinary and Life Sciences, University of Glasgow, 464 Bearsden Road, Glasgow G61 1QH, UK

## Abstract

Lymphoma is the most common haematopoietic malignancy in dogs, but little is known about the aetiology of this heterogeneous group of cancers. In humans, the Epstein–Barr virus (EBV) is associated with several lymphoma subtypes. Recently, it was suggested that EBV or an EBV-like virus is circulating in dogs. We therefore investigated whether EBV, or a novel herpesvirus, is associated with canine lymphoma using both serological and molecular techniques. In an assay designed to detect antibodies to EBV viral capsid antigens, 41 % of dogs were positive. Dogs with cancers, including lymphoma, were more frequently positive than controls, but no particular association with B-cell lymphoma was noted. EBV-specific RNA and DNA sequences were not detected in lymphoma tissue by *in situ* hybridization or PCR, and herpesvirus genomes were not detected using multiple degenerate PCR assays with the ability to detect novel herpesviruses. We therefore found no evidence that herpesviruses are directly involved in common types of canine lymphoma although cannot exclude the presence of an EBV-like virus in the canine population.

## Introduction

Lymphoma is one of the most common malignancies in dogs (Merlo *et al.*, 2008) and despite treatment is invariably fatal. Little is known about its aetiology and pathogenesis, hampering efforts to develop new therapies. Lymphomas in dogs and humans are a heterogeneous group of tumours, and the spectrum of subtypes is similar; in both species diffuse large B-cell lymphoma is the most common form. Canine lymphoma has therefore been proposed as a good model for the human disease (Vail & MacEwen, 2000) and parallels between lymphoma in the two species suggest that findings in human non-Hodgkin lymphoma will be relevant to the canine disease. Epstein–Barr virus (EBV) causes a significant minority of human lymphomas, which are mainly of B-cell origin (Young & Rickinson, 2004). Several recent publications suggest that EBV or an EBV-like virus is circulating in dogs (Chiou *et al.*, 2005; Chiu *et al.*, 2013; Huang *et al.*, 2012; Milman *et al.*, 2011) and it is therefore possible that this virus also causes some canine B-cell lymphomas. Identification of an EBV-like virus in canine lymphomas would not only aid classification of these tumours, but would help elucidate the underlying mechanisms of oncogenesis.

EBV (*Human herpesvirus 4*; HHV-4) belongs to the family *Herpesviridae*, subfamily *Gammaherpesvirinae*; members of this subfamily exhibit lymphotropism and are more commonly associated with cancer than other members of the family *Herpesviridae* (Damania, 2004). EBV infection in humans is ubiquitous and generally asymptomatic (Henle *et al.*, 1969). The virus infects epithelial cells and B-cells, and following primary infection establishes a reservoir of infection within the resting memory B-cell pool (Babcock *et al.*, 1998). It is causally associated with a range of lymphoid and epithelial cell tumours, including Hodgkin lymphoma, Burkitt lymphoma, post-transplant lymphoproliferative disease, nasal T/NK-cell lymphoma, and nasopharyngeal and gastric carcinoma (Young & Rickinson, 2004). Although EBV encodes a large number of proteins, transformation is associated with expression of a restricted group of non-structural proteins and RNAs that drive tumour proliferation. These include six EBV nuclear antigens (EBNA1, 2, 3a, 3b, 3c and LP), two latent membrane proteins (LMP1 and 2) and the EBV-encoded RNAs (EBERs). Although not all of these proteins are expressed in all tumours, the EBERs are expressed in all cells latently infected with EBV and all EBV-associated tumours (Rezk & Weiss, 2007). A second human gammaherpesvirus, HHV-8, is causatively associated with Kaposi’s sarcoma (an endothelial tumour) and also rare types of lymphoma, including primary effusion lymphoma (Carbone & Gloghini, 2008). Herpesviruses are generally host-specific, having co-evolved with their native species, and although cross-species transmission is rare, it is often associated with high morbidity and mortality (Tischer & Osterrieder, 2010).

Recent results from both serological and molecular studies suggest that a gammaherpesvirus is circulating in dogs. Using Western blot assays designed to detect antibodies to EBV viral capsid antigens (VCA), Chiou *et al.* (2005) reported positive results in serum from 32 of 36 healthy domestic dogs in Taiwan. Using a PCR assay based on the EBV *Bam*HI W sequence, EBV-specific sequences were detected in 15 of 21 canine blood samples and, in a later study, in 10 of 12 samples from a diverse range of oro-nasal tumours (Chiu *et al.*, 2013). These results suggested that EBV may have been transmitted from human to dog. Milman *et al.* (2011) used an indirect immunofluorescence assay (IFA) to screen serum samples from healthy domestic dogs in the UK and USA for antibodies to EBV VCA, with 43 of 112 and 67 of 104 scoring positive, respectively. Infection of dogs by EBV was not supported by molecular data, with only one of 104 palatine tonsil samples testing positive in an EBV-specific *Bam*HI W PCR assay. A further 50 blood and 33 lymphoma tissue samples were negative. Additional screening was performed with a degenerate PCR assay designed to detect regions of the polymerase gene conserved across multiple herpesviruses, but detected only *Canid herpesvirus 1* (CHV-1), subfamily *Alphaherpesvirinae*, in 13 of 137 samples.

Huang *et al.* (2012) investigated the potential role of EBV, or an EBV-like virus, in canine lymphoma. Using recombinant VCA proteins in an ELISA, they detected reactivity in serum from healthy dogs and dogs with lymphoma, and reported that dogs with B-cell lymphoma had higher antibody titres to EBV VCA than those with T-cell lymphoma. Using a degenerate herpesvirus polymerase gene PCR assay, positive results were obtained in two of three B-cell lymphoma samples. Subsequently, EBV EBNA3C-specific sequences were detected in samples from three of nine B-cell lymphomas. In contrast to the initial studies by Chiou *et al.* (2005), sequencing of products from the degenerate PCR suggested that an EBV-related virus, rather than EBV itself, was present in the canine samples.

Degenerate PCR techniques use areas of conserved amino acid sequence within a protein family to detect unknown family members. Assays based on conserved motifs in the herpesvirus polymerase and glycoprotein B (gB) proteins have been used successfully to identify many novel herpesviruses, including gammaherpesviruses, in a diverse range of species, including those of the orders Primates, Artiodactyla and Carnivora (Ehlers *et al.*, 2008). Analysis of these sequences led to the formation of a new genus, *Percavirus*, within the subfamily *Gammaherpesvirinae*. This genus brings together viruses from several carnivores, including cat and hyena, and it is likely that novel canine gammaherpesviruses would belong to this group.

The aim of this study was to determine whether a herpesvirus, particularly a gammaherpesvirus, is involved in the pathogenesis of canine lymphoma by using both serological and molecular techniques, including degenerate PCR assays, to investigate common types of canine lymphoma.

## Results

### Serological reactivity detected in EBV VCA assays

**Fig. 1.**
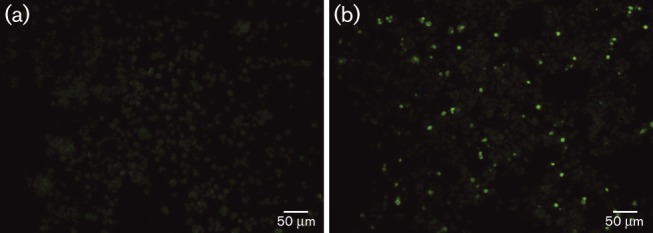
EBV VCA immunofluorescence using canine sera. (a) Negative serum demonstrating dull green background staining. (b) Positive serum with individual fluorescing cells against a duller background.

Serum or plasma samples from 234 dogs were screened using an IFA designed to detect antibodies to EBV VCA (Henle *et al.*, 1969; Milman *et al.*, 2011). In total, 97 samples (41 %) scored positive. Positive canine samples displayed cytoplasmic fluorescence in a proportion of cells, comparable with that seen with human positive controls (Fig. 1). Twenty samples, including 10 that were positive and 10 that were negative by EBV VCA IFA, were tested in a CHV-1 neutralization assay, but none was positive, indicating that positivity did not result from cross-reactivity with CHV-1. This is consistent with the findings of Milman *et al.* (2011). In the combined cancer group, 90 of 195 (46 %) scored positive compared with seven of 39 (18 %) in the control group and differences were statistically significant (χ^2^ test, *P* = 0.001). The proportion of dogs with lymphoma that scored positive (70 of 158; 44 %) was lower than that of dogs with other cancers (20 of 37; 54 %), but this difference was not statistically significant (χ^2^ test, *P* = 0.28). Amongst lymphoma cases with available data (*n* = 115), positivity did not vary significantly by B- or T-cell immunophenotype, sex or age. Antibody titres were similar in dogs with B- and T-cell lymphoma (geometric mean titre 34.09 and 29.97, respectively).

**Fig. 2.**
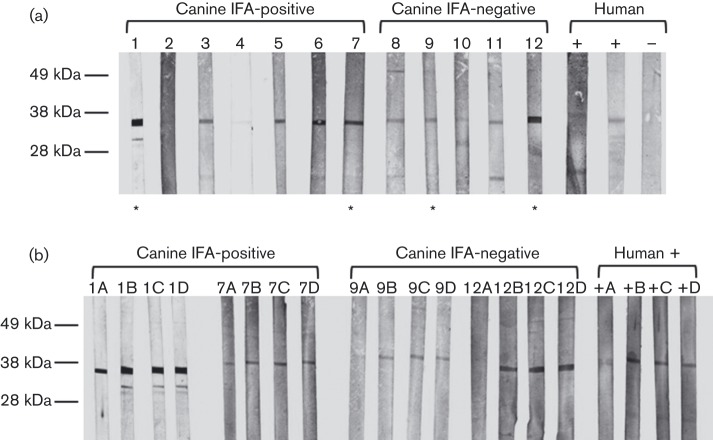
Reactivity with recombinant EBV VCA p18-GST protein in selected canine plasma samples. (a) Canine plasma samples were screened for antibodies to VCA-rp18 by Western blotting. Samples included seven which had tested positive by EBV VCA IFA, five negative by IFA and three human samples, two positive by IFA and one negative. A major band migrating just below the 38 kDa size marker is seen in all samples with the exception of the human negative control. (b) Canine samples 1, 7, 9 and 12, marked with an asterisk in (a), were used in subsequent blocking experiments. These samples, comprising two positive by IFA and two negative, and a human positive sample were pre-incubated with VCA-rp18 (A), FIV p24-GST (B), 10 % BSA (C) or dilution buffer (D) prior to Western blotting. In all samples, there is a reduction in the intensity of the major band in strip A (VCA-rp18) in comparison with the other three strips.

To investigate whether positivity in the IFA assay was specific for EBV VCA, 12 samples were tested for reactivity with a recombinant EBV VCA p18 protein (VCA-rp18) by Western blot analysis. Using IFA-positive human sera as positive controls, a band was observed just below the 38 kDa size marker; this corresponded to the expected size of the glutathione *S*-transferase (GST)-tagged protein (Fig. 2a). No reactivity was detected using the human negative control, but a band of the same size was visible in all canine samples, both IFA-positive and -negative. In both groups, these bands varied in intensity, although with one exception IFA-negative samples gave fainter bands. To further test the specificity of the antibody binding, two samples from each group and a human positive control were pre-incubated with VCA-rp18, a non-related GST-tagged viral protein [feline immunodeficiency virus (FIV) p24], concentrated BSA or dilution buffer prior to Western blot analysis. A reduction in band intensity was seen with all samples in the group pre-incubated with VCA-rp18, but not with samples in other groups (Fig. 2b).

### Quantitative PCR (qPCR) does not detect EBV genomes in canine tissue samples

A qPCR assay to the *Bam*HI W repeat region of EBV was used to screen 111 canine samples, comprising 62 B-cell, 39 T-cell and one null-cell lymphoma, and nine samples from reactive lymphoid or other tumour tissue. The assay was highly sensitive, consistently detecting two copies of EBV in the human cell line Namalwa, which contained one EBV genome per cell. All canine samples were negative.

### *In situ* hybridization (ISH) does not detect EBER RNAs in canine lymphomas

ISH for EBERs was employed to identify canine tissues containing cells latently infected by EBV. Sections from seven B-cell, two T-cell and one null-cell lymphoma were tested. Clear nuclear staining was seen within tumour cells, but not bystander cells in sections from the EBV-associated Hodgkin lymphoma sample, which was used as the positive control. All canine samples were scored negative.

### No evidence of herpesvirus sequences in canine tissue samples by degenerate or ‘canine-specific’ PCR

**Fig. 3.**
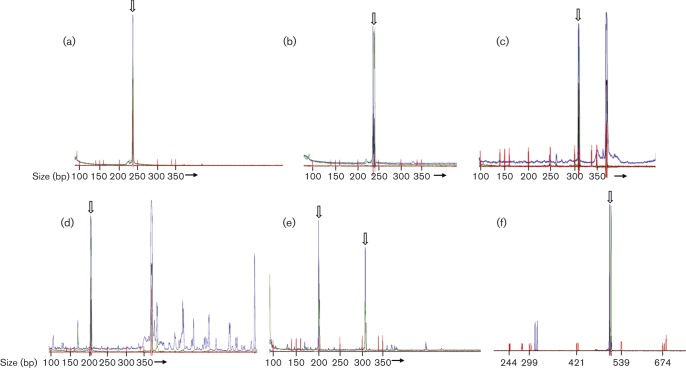
Representative electropherograms from degenerate herpesvirus PCR. Open arrows indicate the described products. Size markers are shown in red with size (bp) indicated below. (a) Semi-nested POL assay. EBV control (250 copies) yields product of 238 bp dual-labelled in blue and green. (b) Semi-nested POL assay. HHV-6B control (iciHHV-6B DNA; 500 copies) yields product of 238 bp dual-labelled in blue and green. (c) Semi-nested POL assay. HCMV control (clinical isolate; 5000 copies) yields product of 313 bp dual-labelled in blue and green. The product >350 bp labelled in blue only is non-specific. (d) Semi-nested POL assay. Canine sample yields product of 200 bp dual-labelled in blue and green; sequencing revealed canine genomic sequence. Products >350 bp labelled in blue only are non-specific. (e) Gammaherpesvirus POL assay. Canine sample yields products dual-labelled in blue and green at ~200 and 310 bp; sequencing revealed canine genomic sequence. (f) gB assay. HHV-8 control (150 copies) yields product of ~500 bp labelled in blue and green.

Samples from 112 canine samples were tested by semi-nested PCR using a degenerate PCR based on conserved sequences in the herpesvirus polymerase protein (‘POL assay’). Samples were from 68 B-cell, 33 T-cell and one null-cell lymphoma, three ‘other tumours’, and seven reactive lymphoid tissues; serum samples from nine patients were positive by EBV VCA IFA and 16 were negative. Analysis of dilutions of control DNA samples demonstrated that this assay could consistently detect 250 copies of EBV, 500 copies of HHV-6B and 5000 copies of human cytomegalovirus (HCMV; HHV-5). Positive controls were positive in all assays and all water controls were negative. The expected amplicon size for known herpesviruses ranges from 238 (EBV, Fig. 3a; HHV-6B, Fig. 3b) to 313 bp (HCMV, Fig. 3c). The 5′ and 3′ primers were labelled with different fluorochromes; therefore, specific products would be labelled with both dyes. Most canine samples yielded products outside the expected size range that were labelled with only a single fluorochrome (Fig. 3d); this indicated that these were non-specific products – an inherent feature of assays incorporating highly degenerate primers.

Seven B-cell lymphoma, two T-cell lymphoma and two reactive tissue samples yielded dual-labelled products. Most were outside the expected size range for herpesvirus amplicons, most commonly ~200 (Fig. 3d) or 325 bp. Products from six samples, ranging in size from 170 to 325 bp and including two samples within the expected size range for herpesviruses, were successfully cloned, sequenced and subjected to blast and blat searches. No matches to herpesvirus sequence were detected. Five amplicons had 99–100 % identity to repeat regions of the canine genome; the final 200 bp amplicon had ~70 % identity, over 50–70 % of its length, to eukaryotic DNA polymerases from a diverse range of species, including *Canis familiaris*.

Alignment of amino acid sequences from the polymerase protein of the recently described genus *Percavirus* demonstrated a mismatch in the pentapeptide sequence (YPSII/M to YPTII) used to design the outer 5′ primer of this assay. As the inner 5′ sequence, based on YGF/VTG, is perfectly conserved amongst gammaherpesviruses, a subset of samples was tested with the inner primer set only (gammaherpesvirus POL assay). These samples comprised 24 B-cell lymphomas, 13 T-cell lymphomas, one null-cell lymphoma and two samples from reactive lymphoid tissue. This single-round assay consistently detected 250 copies of EBV, but no dual-labelled products of the expected size were detected in any of the samples. All water controls were negative. Products of 200 and 310 bp were noted in almost all of the 40 canine samples (Fig. 3e). A 200 and a 310 bp product from a single case, along with a 200 bp product from a separate case were successfully cloned and sequenced. The 200 bp products had identical sequence, which blat searches revealed had 100 % identity to an undefined region of canine chromosome X. The 310 bp product had 100 % identity to a portion of the CCDC57 gene on canine chromosome 9.

The same subset of 40 samples was tested using a degenerate PCR assay based on the gB gene of gammaherpesviruses. This assay consistently detected 150 copies of the HHV-8 genome (Fig. 3f), but no dual-labelled products of the expected size were found in the canine samples. Numerous products outside the expected size range were amplified from all canine samples. All water controls were negative.

**Fig. 4.**
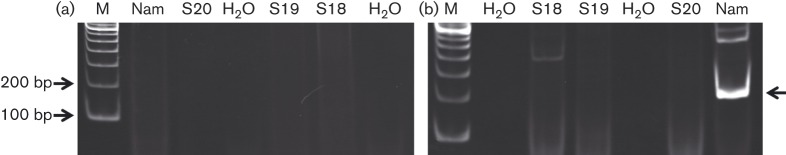
Analysis of canine samples using a gammaherpesvirus-specific PCR assay. Representative gel images. Positions of 100 and 200 bp markers are indicated; the specific product in this assay would be expected to be ~150 bp. M, marker; Nam, positive control (Namalwa); S18-20, canine samples. (a) Tumour DNA as template. No products are detected in sample, water or positive control lanes. (b) PCR products of semi-nested POL assay as template. A band of ~200 bp (arrow) is seen in the positive control lane. Sequencing confirmed that this was derived from EBV. No bands of this size are seen in sample or water lanes.

Huang *et al.* (2012) described a short length of nucleotide sequence, suspected to be a novel canine gammaherpesvirus polymerase gene sequence. Twenty canine B-cell lymphoma samples were tested using primers specific to this sequence. Five samples were from patients who were positive by EBV VCA IFA and seven were from IFA-negative patients. The assay was initially run using 250 ng tumour DNA as template, but no amplification products were detected in either canine samples or Namalwa DNA, used as a positive control (Fig. 4a). The assay was repeated using 2 µl first-round product from the degenerate POL assay as template. No product of the correct size was seen in any of the canine samples, but a clear product of ~200 bp was detected in the positive control (Fig. 4b). This fragment was cloned and sequenced, and corresponded to part of the polymerase gene of EBV. It had been amplified with the 5′ primer from the assay and the 3′ primer from the original degenerate POL assay, which had presumably been carried over from the outer PCR. The putative canine-specific 3′ primer sequence was not present in the amplicon.

## Discussion

In this study, we used serological and molecular techniques to investigate whether a novel canine herpesvirus is directly involved in the pathogenesis of canine lymphoma. The serological data presented here are consistent with the idea that dogs in the UK have been exposed to EBV or a related virus. Over a third of serum samples tested in an EBV IFA – the gold standard for detecting EBV seropositivity in humans – scored positive. The IFA employed in this study used an infected human cell line as the antigen source, raising the possibility that the reactivity resulted from cross-reaction with a protein other than EBV VCA. To confirm assay specificity, samples were analysed by Western blot assay using a recombinant EBV protein (VCA-rp18). In this assay, samples both positive and negative by EBV VCA IFA displayed reactivity, suggesting either greater sensitivity or non-specific binding in the Western blot assay. To address this, antibody was adsorbed by pre-incubation of sera with VCA-rp18. This resulted in a clear and consistent, albeit incomplete, reduction in band intensity; in contrast, no reduction was seen when sera were pre-incubated with a non-related viral protein or a non-specific protein, suggesting at least partial specificity for EBV VCA.

If an EBV-like herpesvirus is involved in the development of canine B-cell lymphoma, then dogs with virus-associated lymphomas would be expected to have higher antibody titres, or a higher prevalence of viral antibodies, than dogs with other conditions. Dogs with cancers, including lymphoma, were more likely to score positive in the IFA than healthy dogs; however, no differences in seropositivity were noted between dogs with lymphoma and those with other cancers, and antibody titres and seroprevalence were similar in dogs with B- and T-cell lymphoma. We therefore did not find any evidence to support a specific association with B-cell lymphoma, although we cannot rule out an association between seropositivity and a subgroup of B-cell lymphomas. Our results could be explained by viral reactivation in sick dogs.

The serology results are broadly in agreement with previously published work, although we detected a lower prevalence of anti-EBV VCA antibodies in our control series than the most comparable study (Milman *et al.*, 2011). Analysis of a larger number of control samples is required to resolve this difference, which may relate to assay sensitivity. Our data on lymphoma patients contrast with those of Huang *et al.* (2012) who reported that dogs with B-cell lymphoma had higher anti-EBV VCA antibody titres than those with T-cell lymphoma or controls in ELISAs. Differences could relate to assay sensitivity or sampling error as numbers were relatively small in both studies.

Taken together, these data suggest that EBV or a closely related virus may be circulating in dogs. This prompted us to perform molecular studies to try and identify the potential agent; as our primary aim was to investigate the aetiology of canine lymphoma, we examined tissue samples with a focus on B-cell lymphoma. We favoured the idea that an EBV-like virus, rather than EBV itself, may be involved as herpesviruses are highly species-specific and rarely cross species barriers (Tischer & Osterrieder, 2010). However, to rule out infection with EBV, we initially screened samples using an EBV-specific qPCR assay based on the EBV *Bam*HI W fragment. This assay detects a repeat region in the viral genome and thus is extremely sensitive, consistently detecting two viral copies per reaction. Analysis of 111 samples revealed no positive results. We also tested for EBV using EBER ISH – the gold standard for determination of EBV positivity within human tumour tissue (Gulley & Tang, 2008). A characteristic pattern of nuclear staining is seen using this assay and it is important to distinguish true-positive staining from non-specific staining, particularly of eosinophils and plasma cells, which is sometimes observed. No positive staining was seen in any of our samples. Although the sample size was small, numbers were similar to other studies. Together, these results make it highly unlikely that EBV is present in, or is involved in, canine lymphoma, despite previous reports that EBV sequences and positive EBER staining are present in canine samples (Chiou *et al.*, 2005; Chiu *et al.*, 2013; Huang *et al.*, 2012).

To determine if another herpesvirus is present in canine tissue samples, multiple degenerate PCR strategies were employed. Such assays are based on amino acid regions that are well-conserved amongst family members and allow the detection of hitherto unknown viruses. Particular effort was made to ensure the assays would detect percaviruses, which include gammaherpesviruses of other carnivores, as it is likely that a novel canine gammaherpesvirus would belong to this group. Similar assays have been used successfully to identify multiple novel gammaherpesviruses, including three recently discovered feline gammaherpesviruses (Ehlers *et al.*, 2008; Troyer *et al.*, 2014). No evidence of herpesvirus infection was found in 112 canine tissue samples using the semi-nested POL assay – the most sensitive of the three assays used. Negative results were also obtained following analysis of a subset of 40 samples using assays targeting sequences conserved in gammaherpesviruses. Sequencing of products in the expected size range of herpesvirus amplicons further confirmed that no herpesvirus sequence was present.

Although degenerate PCRs are less sensitive than standard PCRs, testing of the assays indicated that they had high sensitivity for the control herpesviruses EBV, HHV-6B, HCMV and HHV-8. This level of sensitivity is sufficient to detect viruses present in virally associated tumours, which would be expected to have at least one viral genome per cell. Capillary electrophoresis was used to visualize products as it increases assay sensitivity and, in combination with fluorescent labelling of primers, helps to exclude non-specific amplification, an intrinsic feature of assays using degenerate primers. Stringent measures were observed throughout to avoid PCR contamination and false-positive results.

These results contrast with other studies, where two of three dogs with B-cell lymphoma and 10 of 12 dogs with oral tumours were PCR-positive (Chiu *et al.*, 2013; Huang *et al.*, 2012). This is the largest group of canine lymphoma tissue samples tested to date and thus, by comparison, we expected some positive cases. Huang *et al.* (2012) utilized a similar degenerate polymerase gene PCR to this study, amplifying a product from one canine B-cell lymphoma. They subsequently designed a ‘canine-specific’ primer based on the sequence of this product which was used to identify an additional positive sample. We tested a larger case series with the latter assay, but amplified no products from canine B-cell lymphoma samples. Whilst the PCR data conflict with previously published work, we are confident that we would have detected a virus present in the tumour cells given the number of samples that were analysed and the sensitivity of the assays.

In conclusion, our results provide no evidence that a canine gammaherpesvirus is involved in common subtypes of canine lymphoma. We cannot exclude the possibility that a gammaherpesvirus is involved in rare lymphoma subtypes not included in the current study, analogous to HHV-8 involvement in primary effusion lymphoma. Whilst we did not identify viral sequences in our canine lymphoma samples, it is possible that a canine gammaherpesvirus is circulating in dogs and is present at a low level in lymphoid cells in healthy dogs. Further investigations are required to resolve this issue and determine whether the serological findings reported here and in other studies do indeed reflect infection with an EBV-related virus.

## Methods

### 

#### Patients and samples.

Clinical samples were obtained from untreated patients presenting to the Oncology service at the University of Glasgow Small Animal Hospital, UK or the Centro Veterinario Berna, Portugal. Samples consisted of excess material taken as part of routine evaluations at the time of diagnosis. Ethical approval for their use was obtained from the Faculty of Veterinary Medicine Ethics and Welfare Committee, University of Glasgow. Diagnoses were confirmed by cytology or histopathology. Lymphoma lineage was established by PCR for antigen receptor rearrangements and, where possible, flow cytometry or immunohistochemistry.

Serum or plasma was collected from 195 cases with lymphoma or other tumour types, and 39 healthy controls undergoing routine haematology and biochemistry evaluations prior to use as blood donors (Table 1). All serum and plasma samples were collected from dogs in the UK. The case series included a wide range of breeds; crossbreed and Labrador were the most common, although the proportion of these breeds was similar amongst dogs with lymphoma and dogs with other tumour types. Sex, age and breed information was not available for controls, but all were in the age range 1–8 years and weighed >25 kg. Serum or plasma was separated within 24 h of collection and stored at −80 °C until use.

**Table 1.** Characteristics of patients included in serological analysis All samples were collected in the UK.

Diagnosis	Type*	*N*	Sex (F : FN : M : MN)†	Age (years : months)	
Median	Range
Lymphoma	B-cell	62	8 : 18 : 21 : 15	7 : 0	8 : 0–13 : 8
	T-cell	48	5 : 13 : 16 : 14	7 : 3	1 : 5–12 : 3
	Mixed	1	0 : 1: 0 : 0	7 : 11	7 : 11
	Null-cell	3	0 : 3: 0 : 0	6 : 7	6 : 6–8 : 4
	NOS	44	7 : 9: 18 : 10	7 : 7	1 : 7–16 : 7
Other cancer	Other	37	2 : 17 : 6 :12	8 : 0	3 : 9–13 : 9
Control	Healthy	39	Unknown‡	Unknown‡	1 : 0–8 : 0‡

* NOS, not otherwise specified; patients with ‘Other’ cancers comprised: mast cell tumour (*n* = 12), sarcoma (*n* = 8), osteosarcoma (*n* = 6), carcinoma (*n* = 5), haemangiosarcoma (*n* = 2), and histiocytic tumour, melanoma, neuroectodermal bone tumour and thymoma (*n* = 1 each).

† F, female; FN, female neutered; M, male; MN, male neutered.

‡ The sex and exact age of these patients was not known, although all were aged between 1 and 8 years.

Tissue samples were collected from 125 patients and included fresh material from fine needle aspirates or biopsies (*n* = 112), or formalin-fixed, paraffin-embedded (FFPE) material from biopsies (*n* = 13) (Table 2). Samples were collected from dogs in the UK (*n* = 54) and Portugal (*n* = 71). Most were from lymph nodes; all samples contained a high proportion of lymphoid cells and had a differential diagnosis of lymphoma. A wide range of breeds was represented, with none predominating except for Boxer in the T-cell lymphoma group (nine of 40). Information on neutering status was not available for the Portuguese samples. In the majority of cases, fresh samples were processed within 24 h of collection. DNA was extracted from tissue samples using standard methods and stored at −80 °C until use.

**Table 2.** Characteristics of patients included in molecular analysis

Diagnosis	*N*	Source	Sample type	Site*	Sex (F : FN : M : MN)†	Age (years : months)	
Median	Range
B-cell lymphoma	27	UK	Fresh	LN (18); B (5); AF (1); BM (1); MM (1); SP (1)	2 : 12 : 6 : 7	7 : 6	3 : 0–14 : 0
	41	Portugal	Fresh	LN	20 : 21	10 : 0	4 : 0–17 : 0
	6	Portugal	FFPE	LN	4 : 2	10 : 6	9 : 0–12 : 0
T-cell lymphoma	19	UK	Fresh	LN (9); B (5); BM (1); AH (1)	2 : 9 : 1 : 6	7 : 11	4 : 0–12 : 3
	14	Portugal	Fresh	LN	6 : 7	10 : 0	3 : 0–15 : 0
	7	Portugal	FFPE	LN	3 : 4	11 : 0	4 : 0–15 : 0
Null-cell lymphoma	1	UK	Fresh	LN	0 : 1: 0 : 0	6 : 8	6 : 8
Other	7‡	UK	Fresh	LN (3); BM (1); MM (1); SP (1); TM (1)	0 : 2: 0 : 4	8 : 0	1 : 7–9 : 4
	3§	Portugal	Fresh	LN	0 : 2	7 : 0	5 : 0–9 : 0

* LN, lymph node; B, blood; AF, abdominal fluid; BM, bone marrow; MM, mediastinal mass; SP, spleen; AH, aqueous humour; TM, thyroid mass.

† F, female; FN, female neutered; M, male; MN, male neutered. Information on neutering status was not available for Portuguese samples.

‡ Diagnoses comprised reactive/inflammatory (*n* = 4), and histiocytic tumour, thymoma and thyroid neoplasia (*n* = 1 each).

§ All reactive/inflammatory.

#### EBV VCA IFA.

EBV VCA IFA was performed using P3HR1 cells as described previously (Milman *et al.*, 2011), with minor modifications. To investigate whether this assay detected reactivity with CHV-1, 20 serum samples, including 10 that were positive and 10 that were negative by EBV VCA IFA, were screened for CHV-1 by virus neutralization (Veterinary Diagnostic Services, University of Glasgow). Canine sera were diluted 1 : 10 for initial screening and a 1 : 100 dilution of FITC-conjugated sheep anti-dog IgG (AbD Serotec) was used as the secondary antibody. Human sera diluted 1 : 10 were used as positive and negative controls alongside appropriate control canine sera once identified. Slides were scored independently by two individuals. Where canine sera tested positive, the antibody titre was determined by analysing a twofold dilution series to 1 : 320. The proportion of positive samples in groups was compared and statistical significance determined using χ^2^ analysis implemented in SPSS version 19 (IBM). In the lymphoma group, the effect of immunophenotype, sex and age was assessed using binary logistic regression. Geometric mean titres of samples from dogs with B- and T-cell lymphoma (*n* = 13 and 12, respectively) were compared, and statistical significance of differences in log titre was determined using a two-sample *t*-test.

#### Western blot analysis.

Western blot analysis was performed on 12 canine sera: seven positive and five negative by EBV VCA IFA. Recombinant EBV VCA p18 protein tagged with GST (VCA-rp18; Dundee Cell Products) was resolved on a 12 % polyacrylamide gel and transferred to nitrocellulose membrane. The membrane was cut into strips: each strip contained ~800 ng VCA-rp18. Each strip was incubated with serum or plasma diluted 1 : 20 for 1 h. After washing, the strips were incubated with a 1 : 1000 dilution of biotinylated Protein A (Calbiochem) for 30 min. Reactivity was detected using Vectastain ABC-Amp and BCIP/NBT Substrate kits (Vector) according to the manufacturer’s instructions. Human plasma samples, diluted 1 : 20, that were positive and negative by EBV VCA IFA were used as positive and negative controls, respectively.

Blocking experiments were carried out on four samples: two positive and two negative by EBV VCA IFA. Sera diluted 1 : 20 were pre-incubated with 2 µg VCA-rp18, 5 µg FIV p24-GST (a kind gift from Professor Brian Willett, University of Glasgow) or 10 % BSA for 2 h at room temperature. Western blot analysis was carried out as above.

#### EBV *Bam*HI W qPCR.

qPCR was performed using TaqMan methodology (Applied Biosystems) and primers amplifying the *Bam*HI W repeat region of EBV as described previously (Gallagher *et al.*, 2003) (Table 3). DNA (250 ng) from 98 fresh and 13 FFPE tissue samples was assayed using 1× TaqMan Universal Master Mix without UNG (Applied Biosystems). Tenfold dilutions of DNA from the EBV-positive Namalwa cell line (150 000 to 1.5 copies) were used as a positive control, with water as a negative control after every two samples. Amplification using default parameters for 40 cycles and analysis were performed on a 7500 Real-Time PCR System with Sequence Detection Software version 2.0.6 (Applied Biosystems).

**Table 3.** Primer sequences

Primer	Sequence (5′→3′)*	Reference†
Canine γ-actin 5′	6-FAM-ACCACTGGTATTGTCATGGACTCTG	Gentilini *et al.* (2009)
Canine γ-actin 272 bp 3′	GCTCTTCTCCAGGGAGGACGA	Gentilini *et al.* (2009)
Canine γ-actin 720 bp 3′	TGGCTTTTAGCTCACGGCACC	
POL1A (5′ outer)	HEX-*GACTTTCCAAGTTTC*TAYCCNAGYATHAT	Gallagher *et al.* (2002)
POL1B (5′ outer)	HEX-*GACTTTCCAAGTTTC*TAYCCNTCNATHAT	Gallagher *et al.* (2002)
POL2B (3′ outer and inner)	6-FAM-*TTGATTAAGACGGAG*TCNGTRTCNCCRTA	Jarrett *et al.* (2013)
POL3A (5′ inner)	HEX-*GTTTGATGCCGACCT*TAYGGNTTYACNGG	Gallagher *et al.* (2002)
POL3B (5′ inner)	HEX-*GTTTGATGCCGACCT*TAYGGNGTNACNGG	Gallagher *et al.* (2002)
2760s (gB 5′)	HEX-AAGATCAACCCCACNAGNGTNATG	Ehlers *et al.* (2008)
2761as (gB 3′)	6-FAM-GTGTAGTAGTTGTACTCCCTRAACATNGTYTC	Ehlers *et al.* (2008)
Huang 5′	GGGGTGGCCAACGGCCTCTTT	Huang *et al.* (2012)
Huang 3′	TMMTYCGTAGCTGACTCGGGTGA	Huang *et al.* (2012)
*Bam*HI W 5′	CCCCTGGTATAAAGTGGTCCTG	Gallagher *et al.* (2003)
*Bam*HI W probe	6-FAM-AGCTATTTCTGGTCGCATCAGAGCGC-TAMRA	Gallagher *et al.* (2003)
*Bam*HI W 3′	CCCTCTTACATTTGTGTGGACTCC	Gallagher *et al.* (2003)

* Dye labels are shown where appropriate. Clamp sequences in POL primers are shown in italics. Degenerate bases: Y = C or T; N = A, C, G or T; H = A, C or T; R = A or G; M = A or C.

† References indicate source of previously described primers.

#### EBER ISH.

FFPE tissue sections from 10 UK lymphoma patients were tested for the presence of EBV EBER RNAs as described (Jarrett *et al.*, 2013), using an EBV EBER PNA (peptide nucleic acid)-FITC probe and a PNA ISH Detection kit (both Dako). Samples comprised seven B-cell, two T-cell and one null-cell lymphoma; nine were from lymph node and one was from a gastric mass. The positive control was from a human EBV-associated Hodgkin lymphoma sample.

#### Degenerate PCR assays.

Three different degenerate PCR strategies for identification of novel herpesviruses were used; two of these were based on conserved regions of mammalian herpesvirus polymerase proteins and the third was based on motifs in the gB protein present in many gammaherpesviruses (Ehlers *et al.*, 2008; Gallagher *et al.*, 2002; Jarrett *et al.*, 2013). The primary POL assay was run in a semi-nested format, as described previously (Jarrett *et al.*, 2013). The outer 5′ primers (POL1A and B) were based on the pentapeptide sequence YPSII/M and the inner 5′ primers were based on YGF/VTG (POL3A and B); both outer and inner reactions used a 3′ primer (POL2B) based on the pentapeptide YGDTD. The 5′ primers in both outer and inner primer sets were split into two syntheses to limit degeneracy and increase sensitivity. Each primer also contained a 5′ non-degenerate clamp based on the consensus nucleotide sequence immediately 5′ to the degenerate primer. As it is now apparent that the YPSII/M sequence is not perfectly conserved in all mammalian herpesviruses, particularly gammaherpesviruses, a subset of samples was analysed using only the inner primer set POL3B and POL2B (gammaherpesvirus POL assay). These primers are based on amino acid sequences that are perfectly conserved amongst all gammaherpesvirus protein sequences currently available. The third PCR strategy utilized gB primers that were described previously by Ehlers *et al.* (2008) and used to successfully amplify novel feline gammaherpesviruses (Troyer *et al.*, 2014). The complete series of 112 DNA samples was analysed using the semi-nested POL assay (Table 2), and a subset of 40 samples was analysed using the gammaherpesvirus POL and gB assays.

All PCRs contained 250 ng DNA extracted from fresh tissue or cells, 1× HotStarTaq Plus Master Mix and 1× Q-Solution (Qiagen). Primers (Table 3), labelled with HEX (5′ primers) or 6-FAM (3′ primers), were synthesized by Integrated DNA Technologies and used at a final concentration of 4 µM. In semi-nested reactions, 1 µl first-round product was used as the template. Thermal cycling was performed on a GeneAmp PCR System 9700 (Applied Biosystems); cycling conditions for the first round were: 95 °C for 5 min, followed by 5 cycles of 94 °C for 60 s, 37 °C for 2 min, 72 °C for 3 min, followed by 35 cycles of 94 °C for 60 s, 55 °C for 2 min, 72 °C for 3 min, with a final extension at 72 °C for 7 min. For second-round reactions, and the gammaherpesvirus POL and gB assays, reaction conditions were identical except that a 44 °C annealing temperature was used for the first five cycles.

Sensitivity of the assays was determined by testing dilutions of DNA from: the EBV-positive Namalwa cell line, an individual with inherited chromosomally integrated HHV-6B (iciHHV-6B), a culture of a HCMV clinical isolate (a kind gift from Gavin Wilkie) and the HHV-8-positive BCP1 cell line. Droplet digital PCR was used to accurately determine the number of viral genomes in these samples. The droplet digital assay for HHV-6B has been published (Bell *et al.*, 2014), and previously described assays for EBV, HCMV and HHV-8 (Gallagher *et al.*, 2002) were adapted for droplet digital PCR. Replicates of dilutions containing 50 000, 5000 and 500 genomes of HHV-6B or HCMV and 25 000, 2500 and 250 genomes of EBV were assayed using the POL assay, and 15 000, 1500 and 150 genomes of HHV-8 using the gB assay. DNA from the Namalwa cell line (250 copies) and iciHHV-6B individual (5000 copies) were included in all POL assays as well as plasmids containing the polymerase gene sequence of HHV-6B and HCMV (50 000 copies). For the gB assay, DNA from the BCP1 cell line (150 copies) was used as the positive control. Water was included as a negative control after every two samples. Amplifiability of all samples was assessed using a canine γ-actin PCR amplifying a fragment of 272 bp (Table 3) (Gentilini *et al.*, 2009) with 100 ng DNA per reaction. Cycling conditions were: 95 °C for 5 min, followed by 40 cycles of 95 °C for 30 s, 58 °C for 30 s, 72 °C for 30 s, with a final extension at 72 °C for 30 min. Amplifiability of the 40 samples analysed using the gammaherpesvirus POL and gB assays was assessed as described above using an alternative 3′ primer (Table 3) to amplify a 720 bp fragment.

PCR products were purified to remove excess primer and analysed on a Prism 3130xl Genetic Analyzer (Applied Biosystems). Results were examined using PeakScanner software version 1.0 (Applied Biosystems). Samples generating amplicons in the anticipated size range for herpesviruses, which were dual-labelled with HEX and 6-FAM, were reamplified using unlabelled primers. Products were cloned and sequenced using standard techniques and subjected to blast and blat searches.

#### Gammaherpesvirus-specific PCR.

A subset of 20 B-cell lymphoma samples was assayed using primers described by Huang *et al.* (2012) that were reported to amplify a novel canine gammaherpesvirus sequence (Table 3). Reactions contained 1× HotStarTaq Plus Master Mix (Qiagen) and 250 ng tumour DNA as template. The assay was also performed using 2 µl first-round product from the degenerate POL assay (POL1A, POL2B) as template. Namalwa DNA (2500 copies) was used as a positive control. Thermal cycling conditions were: 95 °C for 5 min followed by 42 cycles of 94 °C for 20 s, 60 °C for 20 s, 72 °C for 45 s and a final extension at 72 °C for 10 min in a GeneAmp PCR System 9700 (Applied Biosystems). Reaction products were analysed by 8 % PAGE. A single fragment of appropriate size was cloned and sequenced using standard techniques.
